# Comparative Aspects of Canine Melanoma

**DOI:** 10.3390/vetsci3010007

**Published:** 2016-02-19

**Authors:** Adriana Tomoko Nishiya, Cristina Oliveira Massoco, Claudia Ronca Felizzola, Eduardo Perlmann, Karen Batschinski, Marcello Vannucci Tedardi, Jéssica Soares Garcia, Priscila Pedra Mendonça, Tarso Felipe Teixeira, Maria Lucia Zaidan Dagli

**Affiliations:** 1 Department of Pathology, School of Veterinary Medicine and Animal Science, University of São Paulo, Av. Prof. Dr. Orlando Marques de Paiva, 87, CEP 05508-270, São Paulo, Brazil; adrinishiya@usp.br (A.T.N.); cmassoco@gmail.com (C.O.M.); cfronca@globo.com (C.R.F.); karen.batschinski@gmail.com (K.B.); Marcello.tedardi@usp.br (M.V.T.); jessica.sg@hotmail.com (J.S.G.); 2 Surgical Oncology Rua Antônio Alves Magan, 124, CEP 01251-150, São Paulo, Brazil; 3 Department of Surgery, School of Veterinary Medicine and Animal Science, University of São Paulo, CEP 05508-270, São Paulo, Brazil; perlmann@ig.com.br (E.P.); priscila.pedra@gmail.com (P.P.M.); 4 Pathology Veterinary Medicine, FEPI Itajubá University Center, Dr. Antonio Braga Filho Street, 687, Itajubá, 37.501-002 Minas Gerais, Brazil; tarsofelipe@hotmail.com

**Keywords:** melanoma, canine, cancer, histopathology, therapy

## Abstract

Melanomas are malignant neoplasms originating from melanocytes. They occur in most animal species, but the dog is considered the best animal model for the disease. Melanomas in dogs are most frequently found in the buccal cavity, but the skin, eyes, and digits are other common locations for these neoplasms. The aim of this review is to report etiological, epidemiological, pathological, and molecular aspects of melanomas in dogs. Furthermore, the particular biological behaviors of these tumors in the different body locations are shown. Insights into the therapeutic approaches are described. Surgery, chemotherapy, radiotherapy, immunotherapy, and the outcomes after these treatments are presented. New therapeutic perspectives are also depicted. All efforts are geared toward better characterization and control of malignant melanomas in dogs, for the benefit of these companion animals, and also in an attempt to benefit the treatment of human melanomas.

## 1. Introduction

Melanoma is a malignant neoplasm originating from melanocytes. Melanocytic neoplasms have been described in men and in most domesticated animal species, including dogs, cats, horses, and also in wild terrestrial and marine animals [1].

Since dogs live in close proximity to humans, they are influenced by similar environmental factors that may lead to chronic diseases and cancer. In fact, the domestic dog (*Canis familiaris*) reportedly presents more naturally inherited chronic diseases than any other animal species. Hence, those aspects have to be considered in the selection of an animal model for a multifactorial human disease, and the dog appears to be the one with the best characteristics [2,3,4]. Dogs are considered recommended models due to their relatively high susceptibility to cancers, including melanomas, and many behavioral similarities [5].

While many genetic alterations of melanomas have recently been discovered and studied, control of this disease is still challenging in both humans and animals, particularly in dogs and cats. The aim of this review is to report a comparative approach of the etiological, epidemiological, pathological, and molecular aspects of melanomas in dogs.

## 2. Canine Melanoma Epidemiology

Melanoma is one of the most devastating types of cancer, killing around 50,000 people worldwide every year [6,7]. In 2015, according to the American Cancer Society, it is estimated that about 73,870 new melanoma cases will be diagnosed (about 42,670 in men and 31,200 in women), and 9940 people are expected to die of melanoma (about 6640 men and 3300 women).

Human malignant mucosal melanoma of the head and neck is increasing rapidly in incidence in the United States. It is an uncommon and fatal malignancy that does not appear to have the same etiologic basis as cutaneous melanoma. In 452 observed cases, the majority of patients had sino-nasal location (72.6%) and white women ages 70 or older were most affected [8]. This kind of human malignant melanoma is uncommon and has aggressive behavior with increasing rates of recurrence and regional and distant metastasis [9]. Regional lymphatic metastasis occurred in 57.9% to 21% of patients and recurrence rates were 57.9% to 26.3% for patients treated with surgery alone and for those treated with surgery and radiotherapy, respectively. Distant metastases occurred in 52.6% receiving surgery alone and in 47.3% receiving both therapies [10].

Canine oral malignant melanomas in dogs accounted for 0.99% of all (338/33826) canine pathology cases received from 1992 through 1999 at the University of Missouri—Veterinary. The most common sites are the gingiva and labial mucosa [11].

In a retrospective study of neoplasms in domestic animals (data collected between 1993 and 2002 at the School of Veterinary Medicine and Animal Science of the University of São Paulo, Brazil), of 1813 cases of neoplasms in dogs, 58 were melanocytic neoplasms, accounting for 3% of the total in this species [12]. In another retrospective study, 2154 neoplasms were diagnosed at the University of São Paulo between 2000 and 2006. A total of 193 cases (8.9%) were melanocytic neoplasms, of which 186 cases occurred in dogs (96.4%), and only seven in cats (3.6%). Male mixed-breed dogs with black hair coats, with ages varying from 8 to 11 years, were the most commonly affected animals [13].

**Figure 1:**
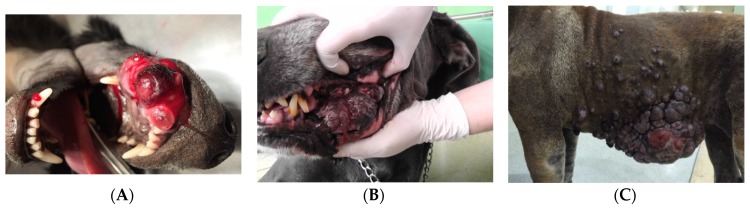
Clinical aspect of malignant oral melanomas (**A**) in maxilla; (**B**) in mandible. Clinical aspect of malignant cutaneous melanoma (**C**) in thoracic and abdominal wall (*Adriana Nihiya*).

Canine malignant melanomas are located at different anatomical sites, such as the mouth (Figure 1A,B), lips, skin (Figure 1C), eyes, and digits (Table 1). Studies are controversial about the most common location for this disease in dogs; however, most studies point to the oral cavity and skin as the most common sites [14,15,16,17]. In a survey of 384 melanocytic canine tumors, oral, cutaneous, lips/feet, and eye sites comprised 19%, 59%, 19%, and 3%, respectively [18]. In another study considering malignant melanomas, oral cavity and cutaneous localization were the most common in 40%–62% and 27%–31%, respectively [13,19].

Data obtained from historical studies indicated that the following breeds of dogs are considered at risk to develop melanocytic tumors: Airedale Terrier, Boston Terrier, Boxer, Chihuahua, Chow Chow, Cocker Spaniel, Doberman Pinscher, English Springer Spaniel, Golden Retriever, Irish Setter [20], Miniature Schnauzer, Scottish Terrier [19,20], Poodles, Beauce Shepherds, Rottweilers, and Labrador Retrievers [19]. Some breeds of dogs, specifically the Schnauzer family (miniature and standard), Scottish Terriers, and Irish Setters are at risk for developing digital malignant melanomas [21,22].

Malignant melanomas typically occur in middle-aged to older dogs, as Miller *et al.* [23] found an average age of nine years, and with no gender predilections.

Teixeira *et al.* [24] studied 25 canine oral melanomas, including 16 melanotic and nine amelanotic specimens. In this study, 25% of the melanotic melanoma-bearing dogs were eight-year old animals, and 22.2% of amelanotic melanoma-bearing group were younger, with ages varying from four to eight years.

Most information on animal cancer epidemiology in the literature comes from laboratories or hospitals, usually from a unique center and using non-standard coding protocols. This may lead to epidemiological bias, and the data rarely generate reliable information useful to establish comparisons. Cancer registries are the most important source of epidemiological data for neoplasms [25]. There are some animal cancer registries around the world, situated mostly in Europe and the USA. In 2013, the São Paulo Animal Cancer Registry was created at the University of São Paulo, Brazil, serving as the first animal cancer registry in Latin America [25,26]. This system will allow for better and more accurate epidemiologic studies in domestic animals in this region.

## 3. Canine Malignant Melanoma Etiology

In humans, malignant melanoma is the most aggressive, therapy-resistant, and deadly form of skin cancer [27]. The major risk factors for human melanoma include family history, skin and mucosal pigmentation characteristics, sun exposure, particularly to UVB light, and skin reactions to sun exposure. Another type of melanoma, oral malignant melanoma, is a rare aggressive neoplasm usually seen in middle-aged adults.

Several etiological factors are supposed to be involved in canine malignant melanomas, including consanguinity, trauma, chemical exposure, hormones, and genetic susceptibility [28]. However, there is no consensus regarding the etiology of malignant melanomas in dogs [29]. Sunlight may be involved in the development of this disease in the sun-exposed skin areas of the body, such as the face and pinnae; however, sunlight probably is not involved in mucosal melanomas, like the ones found in the canine buccal cavity. Other factors, like the presence of pigmented cells, trauma, chemical agents, or even the buccal microbiota, and inflammation may be associated with the etiology of these tumors [30].

Although melanomas can arise in any dog, the prevalence of this disease is higher in purebred dogs, especially Standard and Miniature Schnauzers, Doberman Pinschers, Scottish Terriers, Irish and Gordon Setters, and Golden Retrievers. This fact supports the theory that melanomas in dogs could have a genetic basis [28]. Genetic and epigenetic modifications in melanocytes that lead to alterations in expression or function of genes and proteins involved in cell cycle control and apoptosis are certainly involved in the development of melanomas.

## 4. Canine Melanoma Pathological Aspects and Prognosis

Melanocytic neoplasms can be classified as benign or malignant tumors [31]. Malignant melanomas are considered malignant neoplasms arising from melanocytes, while melanocytomas are the benign counterparts [31].

Melanocytes, which are dendritic cells derived from the neuroectoderm and melanoblasts of the neural crest, migrate during embryogenesis to the dermis and epidermis, mucous membranes, and eyes [29,32]. These dendritic cells in melanoma development have demonstrated altered expression of cell–cell adhesion molecules, such as decreased expression of E-cadherin and V-CAM 1 and increased expression of N-cadherin, Mel-CAM 1, ICAM 1, and α β integrins [33]. The development of malignant melanoma is generally characterized by a series of transitions that are outlined in [34], and arises from melanocytes that normally reside within the basal layer of epidermis [35].

**Figure 2:**
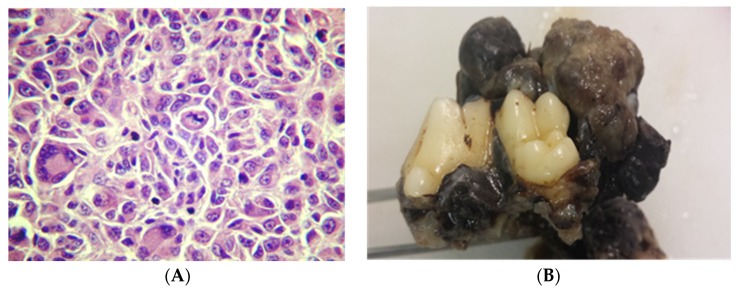
Photomicrograph of the most common histopathology type of malignant melanoma (epithelioid), showing formation of cell nests, with the presence of multinucleated giant cells and atypical mitotic figures, HE, Objective 40 × (**A**). Canine oral malignant melanoma, pigmented, exophytic mass protruding from the gingiva (**B**). Formalin-fixed tissue. Photographs are courtesy of Professor Dr. José Guilherme Xavier.

According to Head *et al.* [36], a melanocytoma is a benign tumor composed of melanocytes in the basal region of the epithelium and extending into the submucosa (junctional melanocytoma) or in the sub-epithelial connective tissue under normal epithelium (dermal melanocytoma). Malignant melanoma can be subdivided into three patterns on the basis of cell shape [36]:

- epithelioid—round and polygonal cells (Figure 2)
- spindle cell—tumor resembles fibroblasts
- mixed tumors—show both cell types

**Figure 3:**
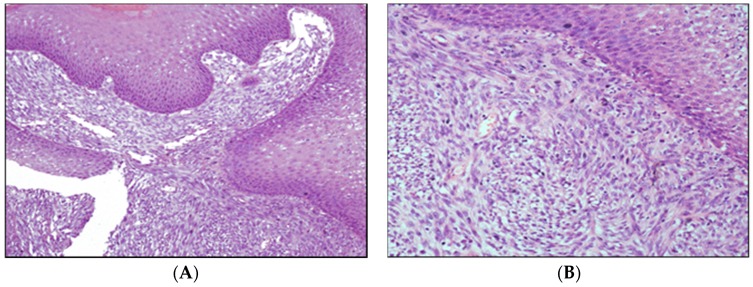
Photomicrograph of oral amelanotic melanoma ((**A**), Objective 10 ×; (**B**), Objective 20 ×). Hematoxylin and eosin.

Malignant melanoma histologically can be the highly melanotic type or the highly anaplastic amelanotic type. Anaplastic melanocytes can be large with abundant cytoplasm with one or more oval or elongated nuclei, with obvious nucleoli. These are frequently characterized to form nests in the submucosa by a mixed structure of epithelial-like cells and fusiform cells and junctional infiltration between basal cells and in the submucosa [36] (Figure 3).

The most prominent biological property of melanoma cells is the ability to produce melanin [37]. The disruption of homeostasis of close association between melanocytes and basal keratinocytes may trigger a continuous proliferation of the melanocytes, which may lead to the development of a malignant melanoma [7]. Once malignant melanoma cells have escaped from the keratinocyte control, they become able to invade the tissue by the degradation of extracellular matrix through the action of metalloproteinases [38].

The histopathological diagnosis of melanoma may be difficult if the tumor does not contain melanin. Amelanotic malignant melanomas may represent one third of all melanoma cases in dogs.

Their histopathological aspect may resemble carcinomas, sarcomas, lymphomas, and osteogenic tumors. For this reason, the diagnosis of malignant melanoma should be made with the use of immunohistochemistry [11,39,40]. Teixeira *et al.* [13] used two different antibodies to confirm the diagnosis of malignant melanoma in amelanotic cases: HMB 45 (Mouse monoclonal anti-human HMB45, from Dako) and Melan A (Monoclonal mouse anti-human Melan-A, from Dako), and both were considered suitable to immunostain canine melanocytes. Ramos-Vara *et al.* [11] considered Melan A as a specific and sensitive marker for malignant melanomas. Smedley *et al.* [41] evaluated 49 amelanotic melanomas and created a cost-effective and efficient immunodiagnostic cocktail panel containing antibodies against PNL2, Melan-A, TRP-1, and TRP-2 that had 100% specificity and 93.9% sensitivity in identifying canine oral amelanotic melanocytic neoplasms. In addition, malignant melanomas are positive for vimentin and variably positive for S-100 protein and neuron-specific enolase (NSE) [42].

In order to use immunohistochemical techniques to study melanoma cell characteristics, it is frequently necessary to remove melanin from the histopathological specimen. There are several methods to do bleaching in melanoma cells, but the one described by Silva *et al.* [43] is one of the most effective, in which slides were immersed only in 10% hydrogen peroxide in pH 7.4 0.2 mol/L Tris-HCl buffer solution for 24 h in the dark at room temperature.

## 5. Canine Melanoma: Molecular Aspects

Melanomas in humans seem to arise from normal melanocytes that suffer a number of molecular events that lead to their transformation. The progression from normal melanocytes to melanoma sometimes involves its transformation to melanocytic nevus. The genetic alterations involved in this progression are still under study. Several genes/genetic pathways were identified in human melanomas, and these are important for the diagnosis, establishment of prognosis, and drug targeting [44]. Some of these genes are described below:

- CDKN2A Locus—about 70% of melanomas harbor mutations or deletions in this locus on chromosome 9p21.
- Genes that are altered when normal melanocytes generate a nevus: BRAF, NRAS, and INK4a/ARF.
- Genes that are mutated or deregulated when a nevus is transformed to a melanoma: BRAF, NRAS, INK4a/ARF, PTEN, c-kit, NEDD9, and MITF.

Genetic alterations in canine malignant melanomas from mucosal or acral sites have not been fully described. Activating mutations in BRAF exon 15 have not been found [6,45]. Activating mutations of NRAS and c-kit appear to be absent in canine mucosal melanoma, in contrast to human mucosal melanoma, where these genes are mutated in 15% of tumors [44,46,47]. Further studies are necessary to understand the molecular basis of canine malignant melanomas.

Genetic alterations in canine malignant melanomas from human mucosal melanomas have not been fully studied (Table 2). Mutations in exon 15 of BRAF are not found [6], similar to human mucosal malignant melanoma [48]. Activating mutations of NRAS are presented in three of 77 cases [19] and c-kit appears absent in canine mucosal melanomas [49], in contrast to human mucosal melanoma, where these genes are mutated in 4% and 14% of tumors, respectively [50].

In 2014, Gillard *et al.* [19] compared the histopathology and genetic alterations of canine and human melanomas. They observed that dog malignant melanomas present cytological characteristics similar to human melanoma simulating “nevus” and “animal type,” suggesting a new melanoma classification. They also studied six genes in canine melanomas that are recurrent mutations in human melanomas, BRAF, NRAS, PTEN, KIT, GNAQ, and CDK4. In total, out of the 95 cases (77 oral and 18 cutaneous melanomas), no mutations were found in BRAF, KIT, GNAQ, and CDK4 genes; only three had a positive NRAS mutation and two had a PTEN gene mutation, of which one case had both changes [19]. BRAF mutations were seen in 57% of human cutaneous melanomas and NRAS was mutated in 17% of samples. Both changes were seen in 15.4% [51].

The low frequency of BRAF mutations associated with UV-independent carcinogenesis and oral anatomical distribution of canine melanomas supports the dog as a spontaneous model for investigation of non-UV-associated human melanomas [52].

Human and canine melanoma share differential gene expression patterns of MAPK, but similar sensitivity in cell culture to therapeutic inhibitors of MAPK (AZD6244) and PI3K/AKT (rapamycin) [6].

Dog malignant melanomas share numerous immunohistochemistry similarities (KIT, PTEN, and phosphorylated forms of AKT and ERK1/2) with human melanoma subtypes; particularly with mucosal, digital and ungual localizations that usually show high growth and aggressive behavior [5].

## 6. Canine Oral Melanomas

Melanoma is considered the most common oral malignancy in dogs [53], accounting for 14.4% to 45.5% of all oral tumors [54,55,56,57,58,59]. Some studies describe melanomas as the second most frequent oral cancer in dogs [54,56]. Oral malignant melanoma may also occur in cats and humans, but it is infrequent [60,61].

Malignant melanoma can be located in any portion of the oral cavity, but the gingival mucosa is the most common site [54,62]. The mandibular labial mucosa was the most common area, affected in 53% of cases, as described by Ramos-Vara *et al.* [11]. Although malignant melanoma rarely affects the tongue, it is considered the most common tumor type of this anatomic site as described by Dennis *et al.* [63], and large-breed dogs, particularly Chow Chow and Shar Pei, are at increased risk for malignant lingual melanoma [62,63].

Small-breed dogs and dogs with pigmented oral mucosa appear to be at increased risk for the development of oral malignant melanoma [61,62]. Cocker Spaniel, Poodle, Pekinese, Gordon Setter, Chow Chow, Golden Retriever, mixed-breed dogs, and Dachshund are the most commonly affected breeds [11,61]. Boxers and German Shepherds appear to be less affected by the disease, as described by Ramos-Vara *et al.* [11].

Dogs with oral malignant melanoma are usually 10.5 to 12 years old, with an average age of 11.4 years [11,54,61]. Males are supposed to be more predisposed to oral malignant melanomas, as verified by Todoroff *et al.* [54] and Vos *et al.* [55]; however, Ramos-Vara *et al.* [11], in a study of 328 dogs diagnosed with oral melanoma, and Teixeira *et al.* [13] found no such predisposition.

**Figure 4:**
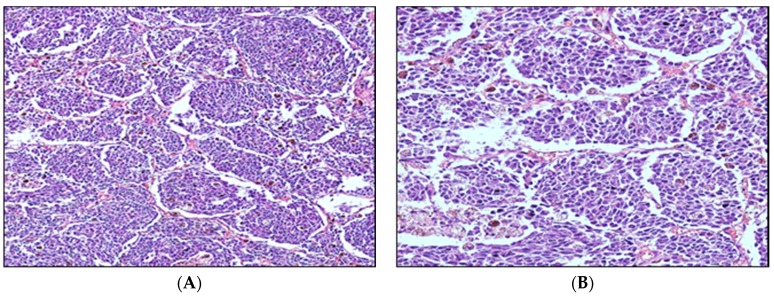
Photomicrograph of lymph node metastasis from oral melanocytic neoplasm ((**A**), Objective 10x; (**B**), Objective 20x). Hematoxylin and eosin.

Oral malignant melanoma is a highly locally aggressive disease, with local invasion into bones occurring in 57% of lesions, with metastasis to regional lymph nodes (Figure 4) in 30.3% to 74% of cases, and to the lungs and other distant organs in 14% to 92% of affected dogs [11,61,64].

Melanoma is one of the few neoplasms in animals for which location is an important prognostic indicator [65]. The statement that all oral melanocytic neoplasms should be considered malignant is commonly found throughout the literature and has become dogma [41].

The prognosis for oral melanoma varies from favorable to unfavorable, with an overall median survival time (MST) of less than 36 months, depending on the intrinsic characteristics of the tumor, and the stage of the disease [61,66,67,68,69].

Historically, many studies confirm that oral and lip melanomas have a poorer prognosis when compared to skin melanomas. The differences concerning the results of different studies of lip melanomas are directly related to the real site of the tumor, that is, either in the skin or in the mucocutaneous junction. In one study, oral melanomas had an MST of 147 days, while the MST of melanoma of the lips and digits were 676 days and 725 days, respectively [18]. Large and poorly or non-pigmented tumors, necrosis, ulceration, high rate of cell proliferation (PCNA expression), and p53 expression are considered poor prognostic factors [29]. Amelanotic melanomas showed a significantly higher amount of mitotic cells when compared to melanotic melanomas [24]. The PCNA (proliferating cell nuclear antigen) in pigmented and non-pigmented melanomas was also assessed. It was demonstrated that cells of all samples stained positive for PCNA, confirming the proliferative activity of this tumor.

The World Health Organization (WHO) staging scheme for dogs with oral melanoma is based on the size of the tumor and the presence of regional and distant metastasis (Table 3) [70]. These staging systems are old and size is not considered a prognostic factor for malignant melanoma [18,28,36], but they are still cited in veterinary practice.

Dogs with stage I melanomas have tumors smaller than 2 cm in diameter, while dogs with stage II and III have tumors measuring between 2 to 4 cm, and more than 4 cm, respectively. Dogs with lymph node metastasis are also classified as stage III disease, and dogs with distant metastasis are classified as stage IV disease. Median survival times for dogs with oral melanoma treated with surgery are approximately 17–18 months, 5–6 months, and 3 months for stage I, II, and III disease, respectively [67].

In a study of 70 dogs with oral melanoma, Tuohy *et al.* [71] observed that 11.4% of dogs had mandibular lymph node metastasis and 1.4% of dogs had lung metastasis at the time of diagnosis. After 821 days (mean time of follow-up), 8.6% of mandibular lymph node metastasis, 17.1% of lung metastasis, 5.7% of metastasis to both sites, and 4.2% of metastasis to lung and other locations were observed. Williams *et al.* [64] reported that 70% of canines with melanomas had metastasis when lymphadenomegaly was present, and that 40% had metastasis in the absence of lymphadenomegaly. Harvey *et al.* [72] observed that dogs with untreated oral melanomas have a median survival time of 65 days.

**Figure 5:**
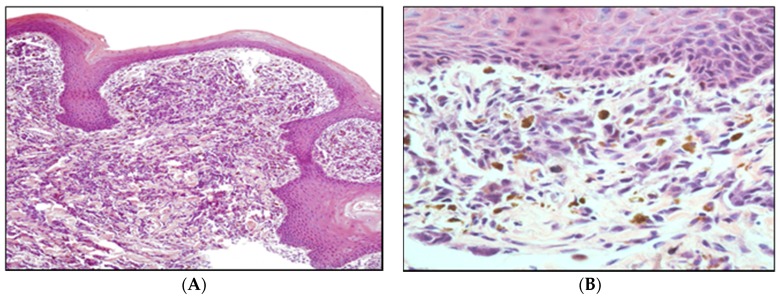
Photomicrograph of oral melanocytic neoplasm ((**A**), Objective 4 ×; (**B**), Objective 40 ×); Hematoxylin and Eosin.

Gillard *et al.* [19] performed clinical and histopathological analyses of 153 malignant canine melanomas with a four-year follow-up. These samples were histopathological characterization by human classification and showed that most canine tumors are intradermal and homologous to human nevocytoid (72%) type, animal type (16.5%), composite type (6.5%), pleomorphic type (4.5%), and superficial spreading melanoma type (0.5%) [19]. Figure 5 shows a representative histology of canine oral malignant melanoma on which local invasion, sparsely pigmented cells, and pleomorphism with variation in shape and cell or nuclear size can be seen.

A recent study of over 382 canine oral melanomas did not find statistically significant differences in survival among different sites or mitotic indices [11].

## 7. Canine Ocular Melanomas

Ocular melanocytic tumors, benign or malignant, may occur in humans and domestic animals. These tumors may affect the eyelids, conjunctiva, orbit, limbo, and uvea [73,74]. Melanomas have different biological behaviors depending on the location in the eye [75]. In general, canine ocular melanomas are less aggressive than oral melanomas. Within the ocular melanoma group, the uveal melanoma is characterized by being more aggressive than the epibulbar melanoma [75].

Eyelid tumors in dogs are usually benign, such as sebaceous adenomas and sebaceous epitheliomas. Among all eyelid tumors, the melanocytic neoplasms account for 20%, and only 8% of these are melanomas [73]. Most, if not all, eyelid skin melanomas are benign [75]. Eyelid melanomas in dogs are usually pedunculated with nodular internal infiltration [73]. They are generally non-aggressive tumors, but occasionally some pigmented melanomas act aggressively and become locally invasive with the development of metastasis [73]. When compared to dogs, melanomas of the eyelid in humans carry a worse prognosis, especially when the tumor is located on the lid margin [76].

Conjunctival melanomas generally affect the nictitating membrane and the bulbar and/or eyelid conjunctiva [77]. Rottweilers and Cockers Spaniels are predisposed [74]. Lesions appear as dark, raised solid masses. Metastasis is rarely seen, but when it occurs it is typically associated with melanomas of the conjunctival eyelid. Complete surgical removal is warranted in early-diagnosed cases [77]. Due to the high risk of local recurrence, enucleation should be considered for invasive tumors [74]. Although conjunctival melanomas in humans are unusual, they have the potential to cause vision impairment and reduce life expectancy, with a mortality rate varying from 18% to 44% [74,78].

Canine and feline limbal melanomas have a benign biological behavior. They appear as raised, well-circumscribed heavily pigmented masses that arise from scleral and subconjunctival connective tissue melanocytes. Most of them develop slowly and are located closely to the superior aspect of the limbus. Labrador Retrievers are genetically predisposed [79].

**Figure 6:**
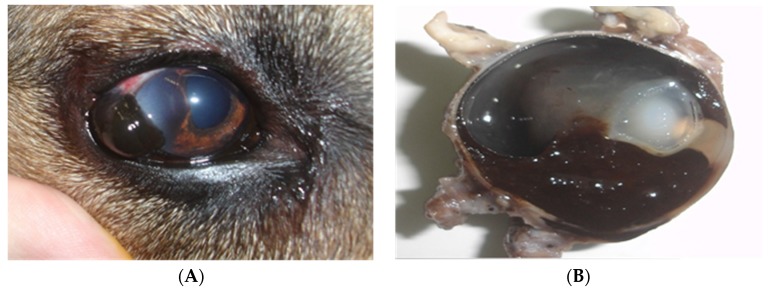
Photographs showing: blackened mass in the iris with extraocular extension (**A**) and sagittal section of the eyeball revealing melanocytic neoplasia of iris, ciliary body, and choroid (**B**) (*Eduardo Perlmann*).

Among the intraocular melanocytic tumors in the dog, melanocytomas are the most common tumors encountered, representing approximately 80% of the neoplasms. Melanomas account for only 20% of tumors [74]. It is difficult and unreliable to clinically differentiate between melanocytomas and melanomas, since melanocytomas can be locally aggressive and even invade the drainage angle and adjacent tissues [73,80]. Therefore, histopathological analysis is indispensable for a definitive diagnosis [81]. Despite the fact that melanocytomas are benign, when located in the iris, they can spontaneously cause necrosis and pigment dispersion, which may lead to obstruction of drainage and cause glaucoma in dogs and humans [82] (Figure 6).

Melanocytomas most commonly affect the anterior uvea in dogs, but rarely affect the choroid [29]. When located in the uvea, they may cause several changes, such as intraocular hemorrhage, uveitis, and secondary glaucoma. Despite the fact that human intraocular melanocytomas are commonly located at the level of the optical disk or adjacent tissues, this location was never described in dogs [74]. An early diagnosis and rapid surgical treatment increase the chances of a favorable prognosis [83]. Wilcock *et al.* [75] studied 91 canine uveal melanocytic neoplasms, and only three developed metastatic disease. These data emphasize the low metastatic rate of uveal melanoma in dogs. Giuliano *et al.* [84], however, showed that animals affected by uveal melanomas had a lower life expectancy when compared with patients diagnosed with uveal melanocytomas. Tumors located in the uvea most frequently are consistent with primary neoplasia; however, animals presenting with intraocular-pigmented tumors should have a complete diagnostic work-up and physical exam to rule out metastasis [85].

In general, when tumors are only confined to the intraocular space, the recommended treatment is enucleation. Exenteration is indicated when the tumor extends to the extraocular space [83]. Other treatment options include iridectomy or diode laser photocoagulation in small-pigmented tumors of the iris [86]. There is no evidence that chemotherapy is effective in the treatment of ocular melanomas [87], but long-term follow-up evaluation would be needed. Ocular malignant melanoma had significantly shorter survival time than benign melanocytomas [84].

## 8. Canine Digital Melanomas

Melanoma is considered the second most common type of cancer of the digits in dogs [22,29,88]. Local invasion is a common feature of digital melanomas and 5%–58% of dogs with this disease have evidence of bone lysis [22,88,89,90]. Tumors of the footpad and interdigital region are less likely to invade into the bone [22,90]. Besides possible local invasion, regional and distant metastasis via lymphatics is frequently noted. It is estimated that 30%–40% of dogs have metastasis at the time of presentation and that even after appropriate local treatment with surgery, subsequent metastasis is frequently seen [22,88].

Due to its aggressive behavior, canine digital melanomas often require digital amputation. The median survival time for dogs without metastasis at the time of diagnosis treated with digital amputation is approximately 12 months, with 42%–57% of dogs surviving one year, and 11%–13% surviving two years [22,88,90,91].

### Canine Cutaneous Melanomas

In contrast to digital melanomas, cutaneous melanomas typically have benign behavior in dogs, with the exception of melanomas that develop on the mucocutaneous junctions [18,92]. They account for 0.8%–2% of all canine cutaneous tumors [64] and are more commonly seen in dogs with heavily pigmented skin. Predisposed breeds include Scottish Terrier, Poodle, Golden Retriever, Dachshund, Cocker Spaniel, Miniature Poodle, Chow Chow, and Gordon Setter [93]. Benign skin melanomas are usually solitary, small, pigmented, firm, and freely moveable over deeper structures. Malignant melanomas tend to be fast-growing tumors, and often are ulcerated, and pigmented (grey, brown, or black) [21].

The most common sites for benign cutaneous melanomas are the face (near the eyelids), trunk, and extremities. Malignant melanoma is found most frequently on the head, ventral abdomen, and scrotum. When metastases occur, lymph nodes, followed by lungs, are the main organs involved [29].

Brockley *et al.* [94] studied 63 dogs diagnosed with melanomas at different locations. Surgery was performed in 24 cutaneous melanomas, with 88% of dogs alive a year after treatment.

Most dogs are cured with complete surgical excision, although some characteristics of the tumor, such as a mitotic index of ≥3/10 HPF, the presence of ≥20% of nuclear atypia, a Ki67 index ≥15%, ulceration, lymphatic invasion, and tumors extending beyond dermis, may have a negative influence on prognosis [18,41,92].

## 9. Treatment Modalities for Canine Melanomas

### 9.1. Surgery

Surgery is the most common and main treatment option for local management of all melanomas, including oral [69], ocular [74], cutaneous, and digital melanomas [90,92]. Due to its high metastatic potential, systemic therapy should also be considered [61,62].

Tuohy *et al.* [71] studied 70 cases of canine oral malignant melanomas treated with curative intent surgery. Surgical resections were done with wide margins, including 2 to 3 cm of bone margins, and 1 cm of soft tissue margins. Histopathological analysis showed that 72.9% of tumors were completely excised, with 10% of these patients developing local tumor recurrence. In this study, dogs that had surgery as their sole treatment option had a progression-free interval (PFI) longer than 567 days, and an MST of 874 days. In a survey performed by Boston *et al.* [69], among wide-margin surgeries performed, 79.3% (73/92) were completely excised based on histologic evaluation, and the recurrence rate was 8.3% (6/73) in this group. The MST of these dogs was 354 days. Esplin [65] studied 61 dogs diagnosed with well-differentiated oral melanomas that were surgically excised, and reported an MST of 34 months with 3.2% of local recurrence.

Regional lymph node resection has been studied in maxillofacial tumors. The analysis of sentinel lymph nodes provides essential information regarding the clinical stage of disease, and also helps determine the appropriate treatment plan [95,96]. As previously shown, in a survey of 100 dogs with oral malignant melanoma, 53% had cytological or histopathological evidence of mandibular lymph node metastasis, even in normal sized nodes [64]. Therefore, regional lymph node removal is warranted, especially in dogs with oral melanomas.

### 9.2. Radiation Therapy

Radiation therapy is effective for malignant melanomas in dogs and may be useful for the control of local disease and involved regional lymph nodes [97]. Response rates are generally best for smaller tumors rather than for salvage therapy against large tumor burdens late in their clinical progression [98]. Another prognostic factor that appears to affect therapy outcome includes the presence of bone lysis. Proulx *et al.* [97] found that dogs without evidence of radiologic bone destruction experienced longer disease-free intervals and overall survival when compared to dogs with bone invasion.

Although melanoma has traditionally been viewed as a “radio-insensitive” disease, many studies suggest a significant role for radiation therapy in the treatment of melanoma [99]. Optimal fractionation schedules have not yet been established for canine malignant melanoma; however, hypofractionated protocols have been utilized with some success [93]. Overall response rates range from 82% to 94.4%, with the complete and partial resolution of tumors in 51% to 69% and 25% to 31% of cases, respectively [91,92,93,94,95,96,97,98,99,100,101,102]. The reported median time to progression ranges from 3.6 to 7.9 months with a median survival time varying from 5.3 to 11.9 months [97,98,100,101,102,103].

Chemotherapy alone has generally offered little benefit in terms of clinical outcome for the control of local canine melanomas; however, a few published studies have shown that the association of radiation with chemotherapy may potentially slow local progression and/or improve overall survival. Cancedda *et al.* [102] compared the efficacy of radiation therapy (5 × 6 Gy) alone and radiation therapy with post-radiation temozolamide (60 mg/m^2^ PO for five consecutive days every 28 days) in dogs with measurable malignant melanoma. Dogs treated with temozolamide had a significantly longer median time to progression (6.8 months) than dogs treated only with radiation (3.6 months). Freeman *et al.* [103] retrospectively looked at dogs with incompletely resected oral melanoma treated with either cisplatin (10–30 mg/m^2^ IV) or carboplatin (90 mg/m^2^ IV) given once weekly 1 h before receiving radiation therapy (6 × 6 Gy). The reported median survival time was 11.9 months, representing the longest survival time when compared to the survival time previously reported for dogs with incompletely resected oral malignant melanoma [103]. Other studies combining radiation with chemotherapy agents, such as carboplatin, cisplatin, or melphalan, did not have a significant impact on either time to progression or overall survival [97,101,104].

### 9.3. Chemotherapy

Despite treating dogs with appropriate local therapies, such as surgery and/or radiation therapy, the ultimate cause of death, especially in oral melanomas, is metastasis. Therefore, the use of systemic therapies is typically recommended. Many chemotherapy protocols have been published in the veterinary literature with minimal improvement in survival times when compared to local treatment alone.

Many chemotherapy protocols using platinum agents have been studied. Rassnick *et al.* [104] reported an overall response rate of 28% using carboplatin as a single agent (300 to 350mg/m^2^/IV/ every 21 days) prior to surgery in dogs with oral (25/27) and cutaneous melanomas (2/27). Boria *et al.* [105] reported an overall response rate of 18% and a MST of 119 days using cisplatin (50 to 55 mg/m^2^/IV/ every three weeks) in combination with piroxicam (0.3 mg/kg/PO/24 hs) in oral melanomas.

Brockley *et al.* 2013 [94] evaluated the effect of carboplatin on the survival of 63 canine patients diagnosed with oral, cutaneous, or digital malignant melanomas. In this survey, carboplatin was administered at 300 mg/m^2^ IV every 21 days for a total of 4–6 cycles. The addition of carboplatin after local therapy did not lead to a significant increase in survival times, and the anatomic site was significantly associated with survival. The overall median survival time for patients with oral, digital, and cutaneous melanomas were 389 days, 1350 days, with a median follow-up of 776 days, respectively.

In another survey, Dank *et al.* [106] compared the outcome of 17 dogs treated with surgery, adjuvant carboplatin (150 to 300 mg/m^2^/IV every three weeks), with or without radiation therapy. The MST was 387 days for the group treated with radiotherapy and 440 days for the group without the addition of radiotherapy.

In a comparative study reported by Tuohy *et al.* [71], surgery with or without any adjuvant therapy (carboplatin, cyclophosphamide as a metronomic therapy, radiotherapy, or xenogeneic canine melanoma vaccine) did not improve overall survival time in dogs with oral melanoma. The MST reported was 874 and 396 days for dogs that had surgery or surgery plus adjuvant therapy, respectively. Boston *et al.* [69] treated 151 dogs with oral melanoma and also concluded that there were no benefits in survival times among dogs that had surgery alone (MST of 335 days for 98 dogs) *versus* dogs that had surgery followed by systemic adjuvant therapy (MST of 352 for 53 dogs). Carboplatin, lomustine, dacarbazine, doxorubicin, metronomic chemotherapy, or commercial melanoma vaccine were the adjuvant therapies used in this study. Postoperative radiation therapy was associated with a longer survival time (MST of 1747 days), but was not significant in the multivariate analysis of this study.

### 9.4. Immunotherapy

Many researchers are currently using and developing therapies to activate the immune system of dogs. The immune responses by therapeutic vaccine represent a potential and powerful strategy for the treatment of melanomas [107]. There is wide evidence that the immune system could modulate the progression and metastasis of melanoma.

Many biological agents have the capacity to demonstrate antitumor activation, such as BCG, IFN-alpha, IL-2, and immunization activators, using an anticancer vaccine [108].

Bacillus Calmette–Guérin (BCG) is used intralesionally in melanomas to activate an immune response and induce tumor regression; however, there may be some complications, such as granulomas and hypersensitivity reactions [109].

*Corynebacterium parvum* is a bacterial substance that has antitumor activity in humans and canines with melanoma. MacEwen *et al.* [67] used *C. parvum* in dogs with stage II and III oral melanomas and compared the outcomes between surgical excision alone and surgery plus *C. parvum* immunotherapy. Animals who received *C. parvum* and surgery had a better prognosis than those with surgery only.

In rodents, dogs, and men, a liposome-encapsulad muramyl tripeptide phosphatidyl ethanolamine (L-MTP-PE), when used *in vivo*, actives monocytes and macrophages, resulting in antitumor activity, which can lead to melanoma metastasis tumor regression or at least stable disease. According to MacEwen *et al.* [108], when L-MTP-PE was used alone or in combination with GM-CSF the antitumor response was minimal, and only prolonged the survival time in dogs with stage I melanoma.

Bianco *et al.* [110], demonstrated that peripheral blood mononuclear cells with apoptotic melanoma cells significantly increased the antitumor immune response, leading to a reduction of the melanoma in three out of five dogs treated intralesionally with FasL-DNA.

Alexander *et al.* [111] used an allogeneic melanoma vaccine in combination with human a glycoprotein 100 (hgp 100), which is a xenogeneic melanoma protein. The vaccine was well tolerated in dogs, and the tumor response rate was 35% for six weeks (including complete response and stable disease).

Hogge *et al.* [112] used an autologous vaccine made of lysate cells transfected with GM-CSF in dogs with soft tissue sarcoma or melanoma. There was a large concentration of inflammatory cells at the site of vaccine administration, different from the ones that received a vaccine placebo only.

A xenogeneic human DNA-encoding tyrosinase protein vaccine (Oncept^®^) has been developed and used in dogs with stage II and III oral malignant melanoma [113]. This vaccine was generally well tolerated in a study of 111 dogs. No systemic reactions occurred, only local hematomas and pain at the injection site [114]. In another retrospective study, medical records from 45 dogs were reviewed, including 30 dogs with stage II and III disease, treated with the Oncept^®^ vaccine after appropriate achievement of loco-regional cancer control. The review found that dogs that received the vaccine did not achieve a greater progression-free survival, disease-free interval, or median survival time than dogs that did not receive the vaccine [115].

The role of dendritic cells (DCs) as a biological adjuvant against malignant melanoma through the induction of an active immune response in the oncologic patient has been explored. Dendritic cells, as professional antigen-presenting cells with an ability to initiate the immune response, could therefore qualify for vaccination strategies to treat human or canine cancers. Compared with the human clinical trials using DC-based vaccines, reports of testing in dogs are scarce.

For humans, at least 13 clinical trials conducted in the United States have been registered using *ex vivo* autologous DCs [116] and, for dogs, only a few reports have been published to strengthen the rational use of DC vaccination for canine malignant melanoma [117,118]. Both protocols used bone marrow-derived DC of dogs with or without malignant melanoma. Gyorffy *et al.* [117] conducted a study using a few canine melanoma bearers and employed DC transduced with an adenovirus vector encoding a xenoantigen, human melanoma antigen gp100, as a coadjuvant of radiation therapy. One dog had an antigen-specific cytotoxic T lymphocyte (CTL) activity in peripheral blood lymphocytes, with no clinical signs, either locally or systemically, with melanoma recurrence 48 months after initial DC injection. However, another dog, which had a negative CTL activity, relapsed 22 months after vaccination. Tamura *et al.* [118], using an autologous DC pulsed with canine melanoma CMM2 cell lysate, efficiently elicited T cell-mediated immunity against CMM-2 cells *in vivo*, reinforcing the rationale to use DC as a therapeutic vaccine.

Although the immunotherapeutic approach using a DC vaccine to drive tumor-specific immune responses appears to be a promising strategy to control malignant melanoma, there are limitations, such as the cost of vaccine production for veterinary use as well as the very fragmentary designed strategies available.

### 9.5. Outcomes after Treatment

Table 4 describes the reported outcomes for canine oral malignant melanoma treated with different protocols.

### 9.6. New Perspectives for the Treatment of Canine Melanomas

The aggressive nature of melanomas in canines and humans, with short survival times and minimal clinical outcome benefits after surgery and adjuvant therapy, emphasizes the importance of new studies to find an effective therapy for treatment and prevention of melanomas metastasis.

A canine therapeutic vaccine has been commercially available since 2007. The Merial melanoma vaccine for dogs (Oncept^®^) consists of DNA encoding the gene for the human melanocyte protein tyrosinase but apparently seems not to cure all melanoma cases. Published studies evaluating the survival times in dogs with melanoma in the mouth undergoing surgery and treatment with the Merial melanoma vaccine report average survival times of 599 days (20 months) or longer [113,114]. These results, though not from randomized studies, are promising and have prompted many veterinary oncologists to recommend the vaccine for patients with stage II and III melanoma despite the lack of FDA-standard clinical trials. The vaccine is well tolerated and has not been reported to be associated with side effects requiring medical intervention. In patients with very large and aggressive melanoma, there may not be sufficient time for an immune response to be mounted against the tyrosinase protein to prevent progression of the melanoma. In fact, 15% of patients treated with the vaccine reportedly die within three months of initiation of treatment, presumably due to the aggressive nature of the melanoma and insufficient time for the vaccine to be effective [119].

Lupeol [120] is among the new therapies under study. Lupeol is a triterpene extracted from various fruits and vegetables that reportedly inhibits melanoma cell proliferation *in vitro* and *in vivo*. A total of 11 dogs bearing buccal melanomas (three, five, and three dogs diagnosed with clinical stage I, II, and III melanoma, respectively) were evaluated. Subcutaneous lupeol (10 mg/kg) was administered postoperatively at various time points. Of the 11 subjects, seven exhibited no local recurrence 180 days postoperatively and no severe adverse effects were observed in any of these cases. Furthermore, no metastases have been detected during the period of the trial.

The current and promising immunotherapy to further augment the options for treatment in malignant melanomas, at least in humans, has been based on checkpoint–blocking antibodies, which can restore anticancer immune responses. Immune checkpoint molecules regulate the immune response avoiding autoimmunity and deleterious effects of an inflammatory response, but when expressed on tumors cells they work as a mechanism of immune evasion. Good examples of immune checkpoint molecules include PD-1 immune checkpoint inhibitor antibody (Pembrolizumab- humanized IgG4; Nivolumab- human IgG4), which was the first drug to gain FDA approval to a phase I trial in patients with BRAF inhibitor-resistant advanced melanoma and BRAFV600 mutation-positive melanoma [121]; and a checkpoint inhibitor (ipilimumab) directed against the cytotoxic T lymphocyte Antigen (CTLA-4). Data from clinical trials has demonstrated the important role this therapeutic approach can play—even with the development of immune-related side effects—since it has been proved a meaningful clinical improvement and an impressive increase in overall survival in melanoma patients [122] when compared to conventional chemotherapy. In the veterinary medicine field, the perspective of this therapy was described by Maekawa *et al.* [123], who demonstrated that an antibovine PD-L1 monoclonal antibody effectively blocked the binding of recombinant PD-1 with PD-L1-expressing tumor cells in a dose-dependent manner, including canine melanoma cells. 

Other therapy as future treatment strategy for canine malignant melanoma can be based on oncolytic virotherapy. Basically, the virus infects and kills tumor cells when replication, lysis, and release of viruses occur. Tumor oncolysis by reovirus infection that was related in Ras-transformed cells was studied in six canine malignant melanoma cell lines CMeC1, CMeC2, KMeC, LMeC, CMGD2, and CMGD5. All of them were susceptible to reovirus infection, although Ras activation was not related in these cells, and only one of them (CMGD2) demonstrated more than 50% of cell death [124].

All efforts are geared toward better characterization of malignant melanomas in dogs, for the benefit of these companion animals, and also in an attempt to benefit the treatment of melanoma in humans.

## 10. Conclusions

Melanomas are very aggressive tumors and dogs are considered good models to study this disease. All efforts are geared toward better characterization and control of malignant melanomas in dogs, for the benefit of these companion animals, and also in an attempt to benefit the treatment of human melanomas. 

## Figures and Tables

**Table 1:** Common sites of malignant melanomas in dogs.

Sites/Total Number of Cases	*n* = 1652	*n* = 143	*n* = 209
Oral	1026	57	67
Cutaneous	448	44	88
Scrotum	-	4	-
Digit	94	21	-
Ungual	60	-	-
Ocular	24	17	-
Lips/Feet	-	-	54
Reference	Gillard *et al.*, 2014 [19]	Teixeira *et al.*, 2010 [13]	Spangler and Kass, 2006 [18]

**Table 2:** Genes altered by mutations in canine and human mucosal malignant melanomas.

	Human	Dog
BRAF	4% [50], 2% [48], 6% [14]	0% [6,19]
C-KIT	12.5% [16], 4% [50]	0% [49]
NRAS	22% [15], 14% [50], 10% [14]	6% [19]
PTEN	48.1% [15]	4% [19]
GNAQ	0% [16]	0% [19]
CDK4	0% [17]	0% [19]

**Table 3:** TNM classification of tumors in domestic animals, WHO, Geneva, 1980.

Clinical Staging System for Oral Tumor			
**Primary Tumor (T)**			
Tis		Tumor *in situ*	
T1		Tumor <2 cm in diameter at greatest dimension	
T1a		Without evidence of bone invasion	
T1b		With evidence of bone invasion	
T2		Tumor 2–4 cm in diameter at greatest dimension	
T2a		Without evidence of bone invasion	
T2b		With evidence of bone invasion	
T3		Tumor >4 cm in diameter at greatest dimension	
T3a		Without evidence of bone invasion	
T3b		With evidence of bone invasion	
**Regional Lymph Nodes (N)**			
N0		No regional lymph node metastasis	
N1		Movable ipsilateral lymph nodes	
N1a		No evidence of lymph mode metastasis	
N1b		Evidence of lymph mode metastasis	
N2		Movable contralateral lymph nodes	
N2a		No evidence of lymph mode metastasis	
N2b		Evidence of lymph mode metastasis	
N3		Fixed lymph nodes	
**Metastasis (M)**			
M0		No distance metastasis	
M1		Distance metastasis	
**Stage Group**	**Tumor (T)**	**Nodes (N)**	**Metastasis (M)**
I	T1	N0, N1a, N2a	M0
II	T2	N0, N1a, N2a	M0
III	T3	N0, N1a, N2a	M0
	Any T	N1b	M0
IV	Any T	N2b, N3	M0
	Any T	Any N	M0

**Table 4:** Outcomes for canine oral malignant melanoma after different therapeutic protocols.

Summary of Treatment Outcome for Canine Oral Malignant Melanoma
**Radiation Therapy (RT)**
- Response Rate = 82%–94.4% - Local Recurrence = 11%–27% - MST (Median Survival Time) = 5.3–11.9 months
***References:*** Theon *et al.*, 1997 (98); Bateman *et al.*, 1994 (100); Murphy *et al.*, 2005 (101); Cancedda *et al.*, 2014 (102); Proulx *et al.*, 2003 (97); Freeman *et al.*, 2003 (103).
**Surgery (SX) with Wide Margins**
- Local Recurrence = 3.2%–10% - MST = 11.8–34 months
***References:*** Tuohy *et al.*, 2014 (71); Boston *et al.*, 2014 (69); Esplin *et al.*, 2008 (65).
**Chemotherapy (CT) for Macroscopic Disease**
- Carboplatin response rate = 28% - Cisplatin + piroxicam response rate = 18%
***References:*** Rassnick *et al.*, 2001 (104); Boria *et al.*, 2004 (105).
**Adequate Local Treatment + Systemic Therapy**
- SX ± RT + adjuvant carboplatin: MST = 14.6 months - SX ± RT + adjuvant carboplatin, metronomic therapy, or melanoma vaccine: MST = 13.2 months (adjuvant therapy did not improve overall MST) - SX + adjuvant carboplatin, lomustine, dacarbazine, doxorubicin, or melanoma vaccine: MST = 11.7 months (adjuvant therapy did not improve overall MST) - RT + post-RT temozolamide: CT did not improve MST, but was associated with a longer median time to progression when compared to dogs treated with RT alone (6.8 months *versus* 2.6 months) - SX + RT + cisplatin or carboplatin once weekly 1 h before RT: MST = 11.9 months
***References:*** Dank *et al.*, 2012 (106); Tuohy *et al.*, 2014 (71); Boston *et al.*, 2014 (69); Cancedda *et al.*, 2014 (102); Freeman *et al.*, 2003 (103).

Abbreviations: RT = radiotherapy, SX = surgery, CT = chemotherapy, MST = median survival time.
